# RNA Virosphere in a Marine Zooplankton Community in the Subtropical Western North Pacific

**DOI:** 10.1264/jsme2.ME21066

**Published:** 2022-01-01

**Authors:** Junya Hirai, Syun-ichi Urayama, Yoshiro Takaki, Miho Hirai, Keizo Nagasaki, Takuro Nunoura

**Affiliations:** 1 Atmosphere and Ocean Research Institute, The University of Tokyo, 5–1–5 Kashiwanoha, Kashiwa, Chiba 277–8564, Japan; 2 Laboratory of Fungal Interaction and Molecular Biology (donated by IFO), Department of Life and Environmental Sciences, University of Tsukuba, 1–1–1 Tennodai, Tsukuba, Ibaraki 305–8577, Japan; 3 Microbiology Research Center for Sustainability (MiCS), University of Tsukuba, 1–1–1 Tennodai, Tsukuba, Ibaraki 305–8577, Japan; 4 Research Center for Bioscience and Nanoscience (CeBN), Japan Agency for Marine-Earth Science and Technology (JAMSTEC), 2–15 Natsushima-cho, Yokosuka, Kanagawa 237–0061, Japan; 5 Super-cuttingedge Grand and Advanced Research (SUGAR) Program, JAMSTEC, 2–15 Natsushima-cho, Yokosuka, Kanagawa 237–0061, Japan; 6 Faculty of Science and Technology, Kochi University, 200 Monobe Otsu, Nankoku, Kochi 783–8502, Japan

**Keywords:** zooplankton, RNA virus, diversity, FLDS, western North Pacific

## Abstract

Zooplankton and viruses play a key role in marine ecosystems; however, their interactions have not been examined in detail. In the present study, the diversity of viruses associated with zooplankton collected using a plankton net (mesh size: 100‍ ‍μm) in the subtropical western North Pacific was investigated by fragmented and primer ligated dsRNA sequencing. We obtained 21 and 168 operational taxonomic units (OTUs) of ssRNA and dsRNA viruses, respectively, containing RNA-dependent RNA polymerase (RdRp). These OTUs presented average amino acid similarities of 43.5 and 44.0% to the RdRp genes of known viruses in ssRNA viruses and dsRNA viruses, respectively. Dominant OTUs mainly belonged to narna-like and picorna-like ssRNA viruses and chryso-like, partiti-like, picobirna-like, reo-like, and toti-like dsRNA viruses. Phylogenetic ana­lyses of the RdRp gene revealed that OTUs were phylogenetically diverse and clustered into distinct clades from known viral groups. The community structure of the same zooplankton sample was investigated using small subunit (SSU) rRNA sequences assembled from the metatranscriptome of single-stranded RNA. More than 90% of the sequence reads were derived from metazoan zooplankton; copepods comprised approximately 70% of the sequence reads. Although this ana­lysis provided no direct evidence of the host species of RNA viruses, these dominant zooplankton are expected to be associated with the RNA viruses detected in the present study. The present results indicate that zooplankton function as a reservoir of diverse RNA viruses and suggest that investigations of zooplankton viruses will provide a more detailed understanding of the role of viruses in marine ecosystems.

Marine viruses are the most abundant “life forms” in the ocean, and they may infect all marine organisms from microbes to mammals (Suttle, 2005). Viral infection, one of the primary causes of the mortality of marine bacteria and phytoplankton, has a major impact on food web structures and geochemical cycles in the ocean (Proctor and Fuhrman, 1990; Suttle *et al.*, 1990). However, the majority of studies on marine viruses have focused on DNA viruses, even though the oceans may also be reservoirs for a vast number of RNA viruses (Steward *et al.*, 2013). In addition, marine viruses infecting meso-sized zooplankton (0.2–20‍ ‍mm) are poorly understood, despite the significant role of zooplankton in linking primary producers to higher trophic levels in the oceans (Beaugrand *et al.*, 2003).

Zooplankton may be vectors of viruses that infect phytoplankton (Frada *et al.*, 2014), fish, and shellfish (Kitamura *et al.*, 2003). Moreover, viral infection may be one of the factors related to the high mortality of zooplankton (Mojib *et al.*, 2017). In marine zooplankton, circular ssDNA viruses were initially reported in two coastal copepod species with high infection rates using both molecular and microscopic observations (Dunlap *et al.*, 2013). Other ssDNA viruses have also been detected in ctenophores (Breitbart *et al.*, 2015) as well as in bulk zooplankton samples from estuaries, coastal waters, and the open ocean (Eaglesham and Hewson, 2013). However, limited information is currently available on RNA viruses associated with marine zooplankton.

The diversity of uncultivated RNA viruses has conventionally been investigated using group-specific PCR primers (*e.g.*, Culley *et al.*, 2003). Although the study of RNA viruses is very limited, particularly for non-model organisms, including zooplankton, the development of transcriptomic approaches unexpectedly revealed the presence of 1,445 different RNA viruses in more than 220 invertebrate host species (Shi *et al.*, 2016). In addition, fragmented and primer ligated dsRNA sequencing (FLDS), which may effectively obtain complete sequences of long dsRNA, including dsRNA viruses and replicating intermediates of ssRNA viruses, is a promising method for revealing the RNA virosphere across a wide range of host species (Urayama *et al.*, 2016). FLDS has been applied to the study of RNA viromes associated with diverse organisms, including fungi, invertebrates, and macroalgae (Chiba *et al.*, 2020; Urayama *et al.*, 2020a, 2020b). It has been employed in examinations of highly diverse viromes with more than 800 viral contigs from marine microbes, including prokaryotes and eukaryotes, which were taken from surface waters in the North Pacific and trapped on membrane filters with a pore size of 0.2‍ ‍μm (Urayama *et al.*, 2018).

To understand the ecological impact of viruses on marine zooplankton, it is necessary to unveil the RNA virosphere within the zooplankton community. In the present study, a bulk sample mainly composed of meso-sized zooplankton was collected using a plankton net (mesh size: 100‍ ‍μm) in the subtropical western North Pacific and analyzed using FLDS. The community structure in the same bulk zooplankton sample was also examined using small subunit (SSU) rRNA sequences assembled from the metatranscriptome of single-stranded RNA.

## Materials and Methods

### Sample collection

Zooplankton sampling was performed in the western North Pacific (25°59.5′N, 126°26.8′E) during the KH-16-07 cruise aboard the RV Hakuho-Maru (Japan Agency for Marine-Earth Science and Technology) on December 18, 2016. One bulk zooplankton sample was collected at a depth of 0–200‍ ‍m by a vertical tow using a North Pacific Standard Plankton (NORPAC) net with a 100-μm mesh. After removing seawater on the mesh, bulk zooplankton were immediately frozen in liquid nitrogen in a 2-mm cryovial (approximately 2‍ ‍g of wet weight) and preserved at –80°C.

### Sample preparation for the RNA virus community

FLDS version 2, as described by Urayama *et al.* (2018), was used to reveal the RNA virosphere associated with the zooplankton community. Briefly, the zooplankton community sample was pulverized in liquid nitrogen using a mortar and pestle. Total RNA for FLDS was obtained from part of the pulverized sample using the conventional phenol-chloroform extraction method. dsRNA was purified using the cellulose column chromatography method (Urayama *et al.*, 2015), and DNA and ssRNA were both removed from the sample using DNase I (Invitrogen) and S1 nuclease (Invitrogen). A Covaris S220 ultrasonicator was used for fragmentation, and fragmented dsRNA was purified using a Zymo Clean Gel RNA Recovery Kit (Zymo). The U2 primer was ligated to the 3′ ends of dsRNA using T4 RNA ligase (Takara Bio), and the product was purified using a MinElute Gel Extraction Kit (Qiagen). After denaturation and annealing with the complementary primer of the U2 primer, we performed cDNA synthesis and amplification using a SMARTer RACE 5′/3′ Kit (Takara Bio). Short DNA fragments including the primers were removed using an 80% volume of Agencourt AMPure XP (Beckman Coulter). We then performed cDNA fragmentation, library preparation, and high-throughput sequencing using an Illumina MiSeq platform to obtain 2×300-bp paired-end sequence reads.

### Data processing for the RNA virus community

Quality filtering of raw sequence data, including removal of the Illumina adaptor, cDNA amplification adaptors, and low-quality, low-complexity, and experimentally contaminated sequences, was performed as previously described (Urayama *et al.*, 2018; Hirai *et al.*, 2021). rRNA sequences were removed using SortMeRNA 2.0 (Kopylova *et al.*, 2012). Assembly was conducted using CLC Genomics Workbench version 9.0 as previously described (Urayama *et al.*, 2018).

The sequences of the assembled contigs were compared to the GenBank non-redundant (nr) protein database (downloaded in March 2021) using the sensitive option in DIAMOND (version 0.9.24) BLASTX (Buchfink *et al.*, 2015). Possible viral contigs were retrieved based on best-hit BLASTX results (e-value cut-off: 1×10^–5^). Regarding contigs with BLAST hits to a viral polyprotein or hypothetical protein, the presence of the RNA-dependent RNA polymerase (RdRp) gene was investigated using Pfam 34.0 (Mistry *et al.*, 2021). Unclassified contigs in the initial BLASTX ana­lysis were subjected to further ana­lyses to recover possible viral contigs with RdRp genes using DIAMOND BLASTX searches against the protein sequences of viruses reported by Urayama *et al.* (2018). The nucleotides of contigs with the RdRp gene were clustered into OTUs with 90% sequence identity using CD-HIT-EST (Fu *et al.*, 2012). The RNA virome was examined using qualitative OTU compositions and quantitative read abundance based on sequence coverage. dsRNA and ssRNA virus communities were analyzed separately because of possible biases in FLDS by detecting only replication intermediates for ssRNA viruses (Urayama *et al.*, 2016).

A phylogenetic ana­lysis was performed for the major taxonomic groups of RNA viruses based on the amino acid sequences of the RdRp gene. In addition to the representative sequences of established taxonomic groups of known viruses, which were reported by the International Committee on Taxonomy of Viruses (Walker *et al.*, 2020), we added virus sequences with high similarities to the contigs based on BLASTX in the present study for the phylogenetic ana­lysis. The amino acid sequences of the RdRp genes were aligned in each viral group using MUSCLE (Edgar, 2004) in MEGA 7.0.21 (Kumar *et al.*, 2016), and conserved sequence regions were manually verified. After ambiguous positions were excluded using trimAl version 1.2 (Capella-Gutiérrez *et al.*, 2009) with the gappyout option, the best substitution model for amino acid sequences was selected based on AIC by Aminosan (Tanabe, 2011). Phylogenetic ana­lyses were conducted with 100 bootstrap replicates using a selected substitution model for each taxonomic group on RAxML 8.2.10 (Stamatakis, 2014).

The full-length sequences of viral segments were obtained by evaluating the terminal regions of the contigs using the method described by Hirai *et al.* (2021). Sequence reads after quality filtering were reassembled using the CLC Genomics Workbench and SPAdes genome assembler version 3.15.1 (Bankevich *et al.*, 2012), and merged with the viral contigs obtained above. Sequence reads with adaptor sequences were selected and mapped against the merged viral contigs using Bowtie 2 version 2.3.4.1 (Langmead and Salzberg, 2012) after removing adaptor sequences. Since a higher abundance of reads mapped on both termini was observed in FLDS, both termini were assessed using the Smirnov-Grubbs test (*P*<0.05) to detect outliers, which were not mapped to the 5' end of the contigs. The same ana­lysis was performed for contigs without BLAST hits for viruses, and genome structures were elucidated based on the conserved sequences of terminal sequences (Urayama *et al.*, 2018). In virus genomes with full-length segments, the positions of the ORFs were predicted using the NCBI ORFfinder, and gene positions were predicted using PfamScan.

### Zooplankton community ana­lysis

The community structure in the same zooplankton sample for FLDS was investigated using the conventional RNA sequencing of single-stranded RNA. Total RNA was extracted and purified from a part of the pulverized zooplankton sample described above. Extraction and purification were performed using TRIzol Reagent (Life Technologies), a TRIzol Plus RNA Purification Kit (Invitrogen), and DNase I (Invitrogen), according to the manufacturers’ protocols. Double-stranded cDNA was synthesized with random primers using a PrimeScript Double Strand cDNA Synthesis Kit (Takara Bio), and cDNA was fragmented using the Covaris S220 ultrasonicator. The library for the Illumina system was prepared using KAPA HyperPrep Kit Illumina platforms (Kapa Biosystems), and the quality and quantity of the constructed library were evaluated using an Agilent 2100 bioanalyzer (Agilent Technologies) and KAPA Library Qualification Kit (Kapa Biosystems). Paired-end sequence reads of 2×300 bp were obtained using the Illumina MiSeq platform.

After the quality filtering of raw sequence data and selection of rRNAs, the full lengths of SSU rRNA were reconstructed using the SILVA SSU database (version 123) on EMIRGE 0.61.0 (Miller *et al.*, 2011). The representative sequences of OTUs clustered at 97% similarity were blasted against the NCBI nt database. We classified OTUs into taxonomic groups based on BLAST results. Quantitative data using sequence reads were standardized by the sequence length of the OTUs.

### Accession numbers

Raw Illumina MiSeq data are available in the NCBI BioProject database (accession number PRJDB9590). The full-length sequences of viral segments are available in NCBI GenBank (accession numbers LC651635–LC651651). Other detailed datasets are available in Figshare (https://doi.org/10.6084/m9.figshare.c.5697835.v1), including alignment files for phylogenetic ana­lyses, the sequence files of contigs with RdRp genes, and the sequence files of possible full-length segments of RNA viruses with no BLASTX hits or BLASTX hits to viral genes other than RdRp.

## Results

### RNA virus community

After quality filtering, 163,818 sequence reads were assembled into 2,857 contigs, and 987,185 sequence reads were left unassembled. 194 contigs (22,879 reads) harbored RdRp genes using BLASTX (e-value: <1×10^–5^), and 19 contigs (20,699 reads) showed BLAST hits to other viral genes (*e.g.*, capsid). Genes in other contigs were identified as non-viral genes (671 contigs and 24,509 reads) or unclassified (1,973 contigs and 95,731 reads) in BLASTX ana­lyses. The contigs encoding possible virus RdRps were clustered into 189 OTUs. Among them, the abundance of ssRNA viruses (21 OTUs with 5,460 reads) was markedly lower than that of dsRNA viruses (168 OTUs and 17,419 reads).

**Fig. 1.**
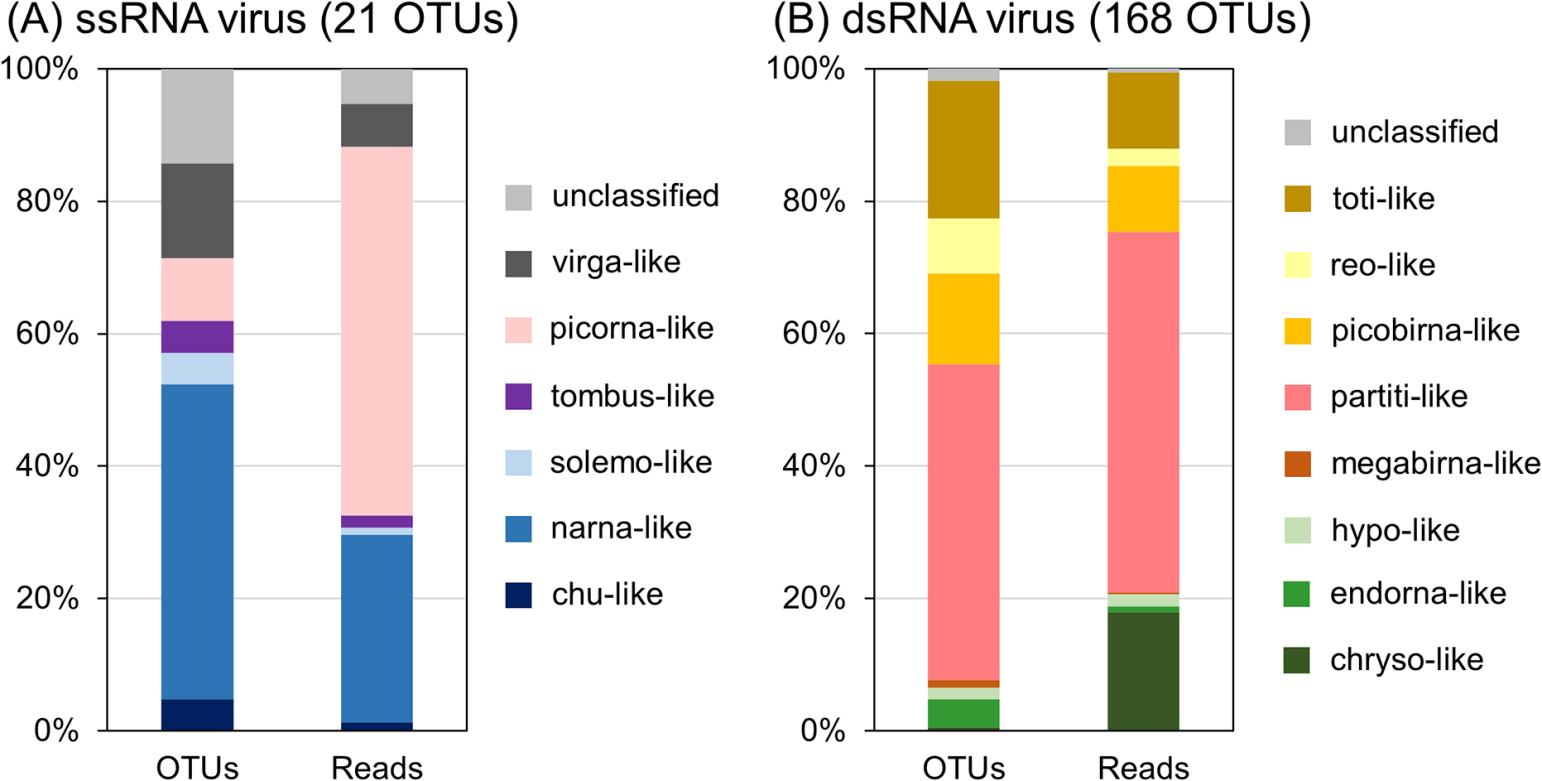
Community structure of (A) ssRNA and (B) dsRNA viruses based on RNA-dependent RNA polymerase (RdRp) gene sequences. The compositions of operational taxonomic units (OTUs) and sequence reads are represented for each RNA virus community.

The OTUs of ssRNA viruses showed significant similarities with the RdRps of six known taxonomic groups and unclassified viruses (Fig. 1A). The highest diversity was found in the OTUs related to narna-like viruses, accounting for approximately half of the OTU numbers in ssRNA viruses. In contrast, picorna-like virus OTU sequence reads were the most abundant (55.7%), followed by those of narna-like (28.3%) and virga-like viruses (6.5%). The proportion of sequence reads for the other viral groups was less than 2% for ssRNA viruses.

The OTUs of the dsRNA viruses were significantly similar to eight groups of known viruses and unclassified viruses (Fig. 1B). OTUs related to partiti-like viruses were the most diverse and abundant group, with 47.6% of OTUs and 54.5% of sequence reads. The second most abundant dsRNA virus OTU group, regarding sequence reads, was that including chryso-like viruses (18.0%); however, this group showed low diversity (<1% of OTUs) among dsRNA viruses. Other major dsRNA virus groups included picobirna-like (13.7% OTUs and 9.9% reads), reo-like (8.3% OTUs and 2.6% reads), and toti-like viruses (20.8% OTUs and 11.5% reads).

### Sequence identity to known viruses

**Fig. 2.**
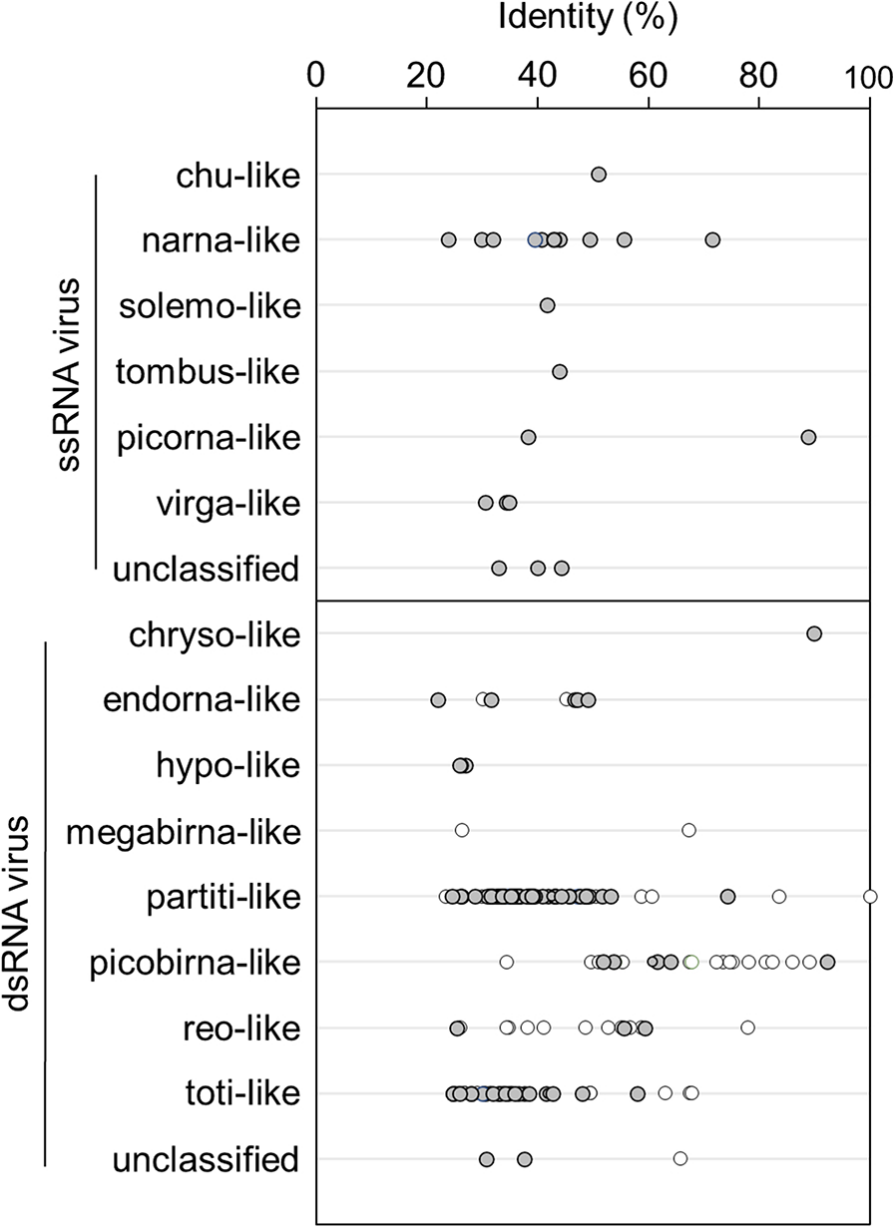
Amino acid identity between RNA-dependent RNA polymerase (RdRp) gene sequences from the present study and those of known viruses. White plot points represent operational taxonomic units (OTUs) with the highest identities to viruses from marine microbes (Urayama *et al.*, 2018).

Most of the RdRp genes obtained in the present study were distinct (≤90% identity) from those of known viruses in Urayama *et al.* (2018) and the GenBank nr protein database (Fig. 2). Only one OTU showed 100% identity with a RdRp of partiti-like viruses obtained from marine microbes (Urayama *et al.*, 2018). Sequence identities to the RdRps of known viruses varied from an average of 33.4% (virga-like) to 63.8% (picorna-like) in ssRNA viruses (average 43.5% identity in all ssRNA viruses). In addition, average identities to the RdRps of known viruses ranged from 26.5% (hypo-like) to 90.0% (chryso-like) in dsRNA viruses (average 44.0% identity in all dsRNA viruses).

### Phylogenetic ana­lyses of major taxonomic groups of RNA viruses

**Fig. 3.**
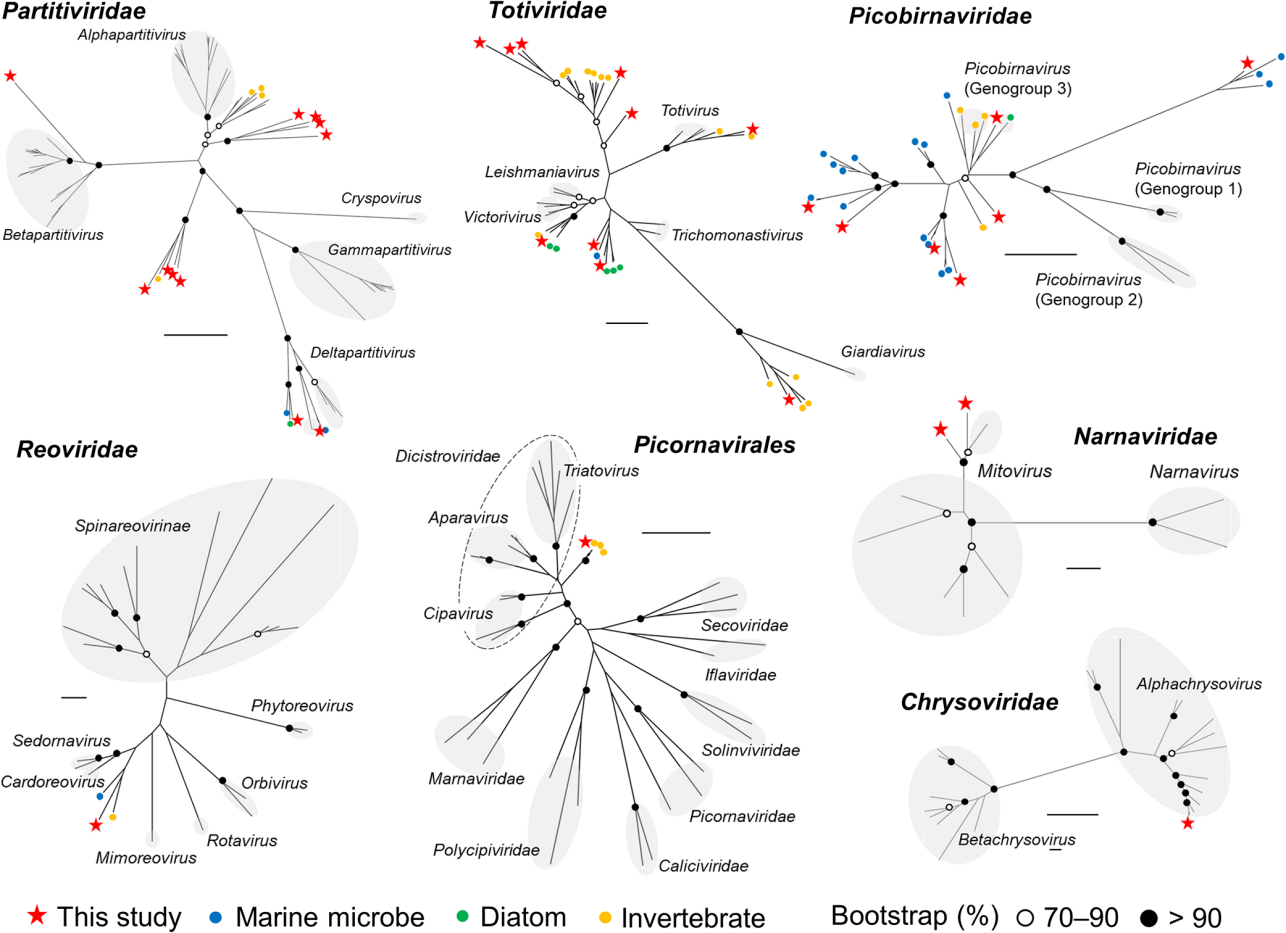
Phylogenetic ana­lyses of major taxonomic groups of RNA viruses. In addition to the RNA viruses detected in the present study, viruses from marine microbes (Urayama *et al.*, 2018) and diatoms (Urayama *et al.*, 2016; Chiba *et al.*, 2020) detected by FLDS and from invertebrates (Shi *et al.*, 2016, 2017; Hahn *et al.*, 2020; Ottati *et al.*, 2020; Yen *et al.*, 2020; Chiapello *et al.*, 2021) detected by a transcriptome ana­lysis are indicated in different colors. Bootstrap values from maximum likelihood ana­lyses are indicated if ≥70%. Scale bars indicate a genetic distance of 0.8. Details on the phylogenetic ana­lysis of each viral group are in the Supplementary materials (Fig. S1, 2, 3, 4, 5, 6, and 7). The best-fitting amino acid substitution models were rtREV+F+G (*Picobirnaviridae* and *Picornavirales*), LG4X+F+G (*Reoviridae*), and LG+F+G (other families).

Phylogenetic ana­lyses of each major taxonomic group of RNA viruses showed that the viruses obtained in the present study were phylogenetically diverse, and most of the OTUs formed new clusters, which were not included in any of the established genera of known viruses (Fig. 3). For example, the OTU included in *Picornavirales* was clustered into a phylogenetic group with other invertebrate viruses. This group was distinct from other established groups of *Picornavirales* viruses and belongs to the family *Dicistroviridae*. Among dsRNA viruses, the OTUs in *Partitiviridae* were clustered into multiple new phylogenetic groups. One of the phylogenetic groups was exclusively composed of OTUs obtained in the present study. Additionally, the other phylogenetic groups consisted of RdRp sequences from marine microbes, diatoms, and invertebrates. The OTUs in the families *Totiviridae*, *Picobirnaviridae*, and *Reoviridae* also formed unestablished phylogenetic groups with viruses from marine microbes, diatoms, and invertebrates. The OTUs in *Chrysoviridae* and *Narnaviridae* belonged to the established groups of *Alphachrysovirus* and *Mitovirus*, respectively.

### Full-length segments of viruses

**Fig. 4.**
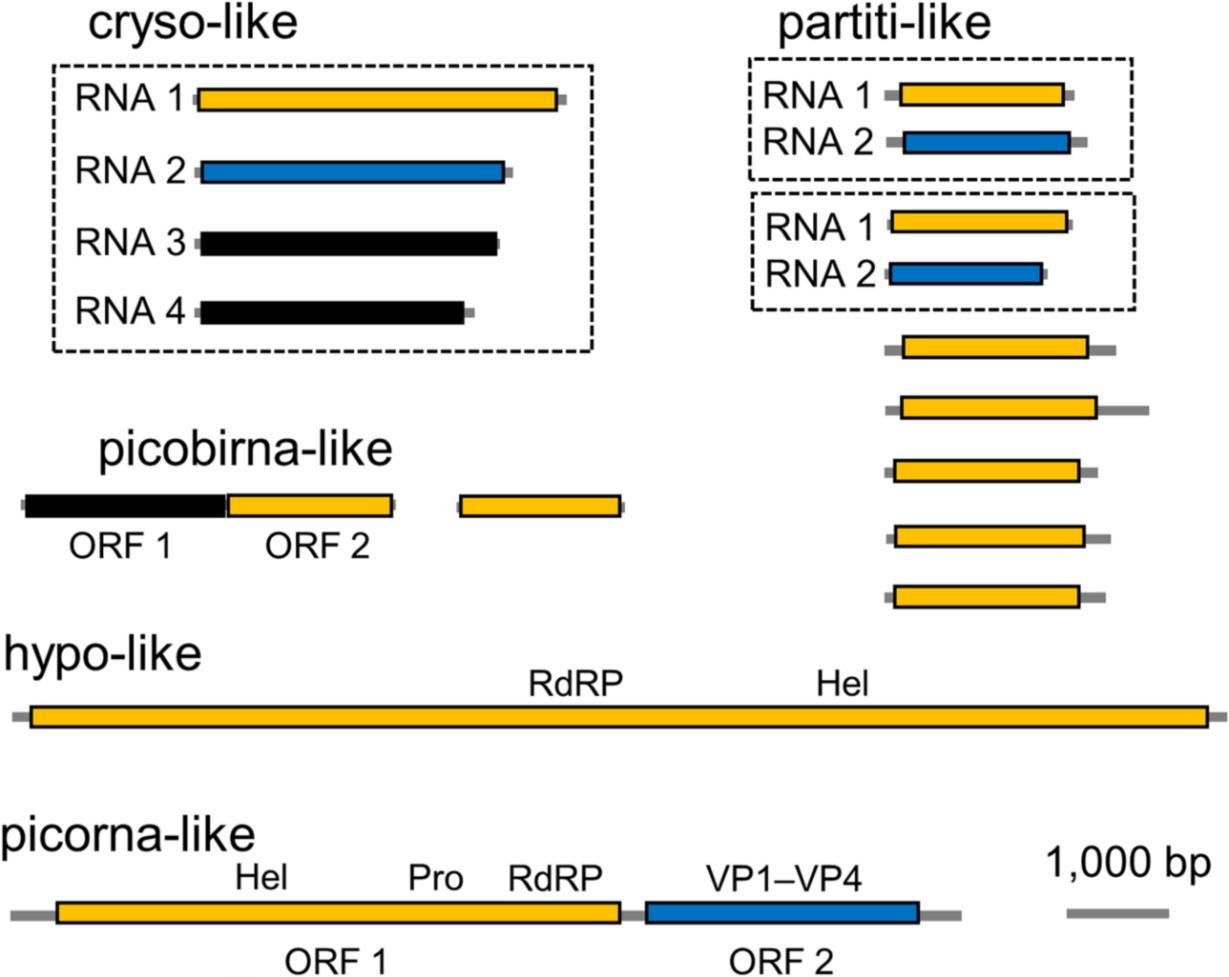
Full-length segments of RNA viruses obtained in the present study. The functions of genes were predicted based on PfamScan. The ORFs including genes encoding RNA-dependent RNA polymerase (RdRp) are in orange, whereas ORFs possibly encoding capsid elements are in blue. Segments derived from the same virus are identified based on the conserved sequences of termini and surrounded by black dashed lines.

FLDS enables the identification of full-length viral segments based on the read mapping of contigs, and sets of the genomic segments of segmented viruses can be identified based on the conserved sequences of the terminal sequences of contigs. One genome of a chryso-like virus (Fig. 4) with four segments (2,813–3,564 bp) was reconstructed. Each segment harbored one ORF, and the RdRp and capsid genes were identified using BLASTX (e-value: <1×10^–5^) in the longest and second-longest segments, respectively. These features were similar to those of chrysoviruses, such as *Penicillium chrysogenum virus* (Jiang and Ghabrial, 2004). In partiti-like viruses that harbor bisegmented genomes (Nibert *et al.*, 2014), seven full-length segments containing the ORF of the RdRp gene (1,811–2,573 bp) were identified. The entire region of the second segment was only recovered in two partiti-like viruses, and one of the two segments showed a BLAST hit to a known *Partitiviridae* capsid gene. Full-length second segments encoding the capsid gene were not found for the other five partiti-like viruses in the present study. The genome of *Picobirnaviridae* is composed of two segments (King *et al.*, 2012); however, a non-segmented genome has also been reported (Urayama *et al.*, 2018). There are two types of full-length segments containing RdRp genes in picobirna-like viruses. One full-length segment (3,648 bp) is composed of two ORFs, and the other segment (1,639 bp) only contains one ORF. Second full-length segments were not found for these two picobirna-like viruses in the present study. The genome of the hypo-like virus (11,845 bp) with an unsegmented genome included one polyprotein ORF containing the RdRp gene. The non-segmented genome of the Picorna-like virus (9,274 bp) harbored two ORFs of the RdRp and capsid-related genes (VP1–VP4).

### Community structure of zooplankton

**Fig. 5.**
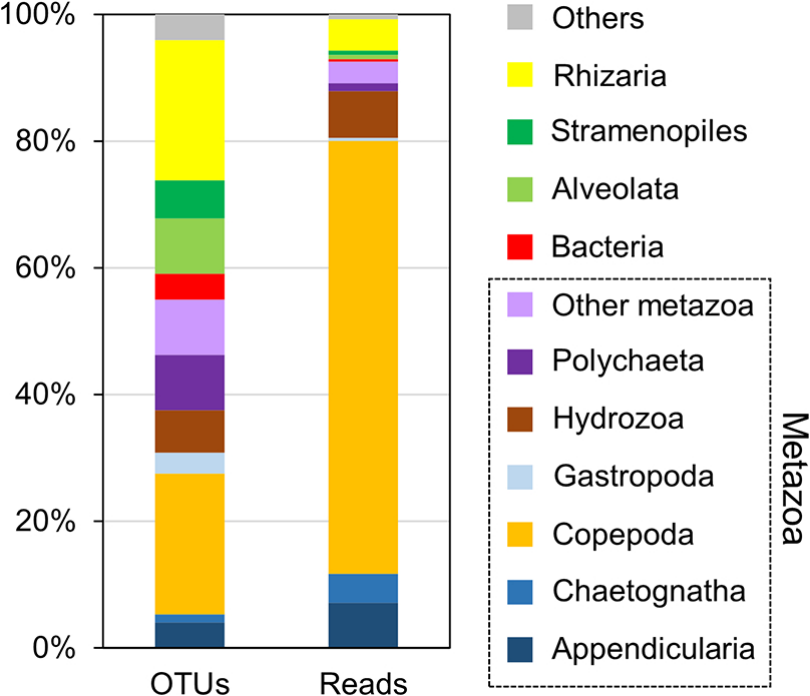
Community structure of zooplankton based on small subunit ribosomal RNA sequences. Proportions of operational taxonomic units (OTUs) and sequence reads are represented for the major taxonomic groups found in the present study. The taxa in Metazoa are surrounded by black dashed lines.

A total of 149 SSU rRNA OTUs were obtained from 53,946 reads (56.4% of total reads in the metatranscriptome of single-stranded RNA), and 82 OTUs were classified as lineages of metazoan zooplankton (Fig. 5). Among them, 33 OTUs belonged to Copepoda, 13 to Polychaeta, and 10 to Hydrozoa (10 OTUs). OTUs belonging to protists, phytoplankton, and bacteria were also detected; 33 OTUs were identified as Rhizaria. In the ana­lysis based on read abundance, Metazoa accounted for >90% of the total reads, with Copepoda being the predominant group accounting for 68.3% of the total reads. The second most abundant taxon was Hydrozoa (7.3%), followed by Appendicularia (7.2%) and Chaetognatha (4.5%). Non-metazoan organisms shared small portions of sequence reads; however, the read abundance of Rhizaria was 5.0%. Among the top 10 dominant OTUs, eight were Copepoda (Table 1). The most dominant OTU, sharing 11.34% of the sequence reads, was classified as a member of the family Clausocalanidae. Other dominant copepod OTUs belonged to the families Paracalanidae, Calanidae, Candaciidae, Oithonidae, and Oncaeidae. OTUs in the families Sagittidae in Chaetognath and Oikopleidae in Appendicularia were the fifth and sixth most dominant OTUs, respectively.

## Discussion

Marine zooplankton and viruses play a key role in marine food web structures and biogeochemistry; however, their interactions have not been examined in detail. In the present study, we used FLDS and successfully obtained the complete and partial genome sequences of RNA viruses, revealing a RNA virome associated with the zooplankton community. The zooplankton community based on SSU rRNA was dominated by metazoan zooplankton, particularly by small copepods, including the families Clausocalanidae, Paracalanidae, Oithonidae, and Oncaeidae, as well as other dominant taxa of Appendicularia, Chaetognatha, and Hydrozoa. These taxa have been reported as abundant zooplankton in the subtropical western North Pacific (Kâ and Hwang, 2011; Tseng *et al.*, 2013; Kobari *et al.*, 2018). The method used in the present study to reveal plankton communities can detect prokaryotes and eukaryotes based on the SSU rRNA gene sequence, and the smaller abundance of non-metazoan taxa was observed. The identification of detailed virus-host relationships based on massive sequence data from environmental communities is challenging (Obbard, 2018); however, the high abundance of Metazoa based on SSU rRNA sequences in the present study suggests that metazoan zooplankton, particularly copepods, function as hosts for RNA viruses. Although ssRNA viruses are underestimated due to the detection of only the replicating intermediates of their genomes using FLDS, the unique community of RNA viruses was considered to be attributed to major zooplankton taxa in the present study.

Collectively, the present results suggest that the zooplankton community acts as a reservoir of diverse RNA viruses because we detected 189 OTUs encoding RdRp, which are mostly new RNA viruses related to 15 taxonomic groups and unclassified RNA viruses. Our study area is in the subtropical western North Pacific, in which a high diversity of zooplankton has been observed (Tittensor *et al.*, 2014). Therefore, the high genetic diversity of RNA viruses may be attributed to the zooplankton community that covers various taxonomic groups in Metazoa. However, only some of the viromes associated with zooplankton were revealed in the present study. We only analyzed a single zooplankton community sample with approximately 2‍ ‍g wet weight, containing 82 SSU rRNA gene OTUs of metazoan zooplankton, while diverse zooplankton species have been reported in this study area (Shih and Chiu, 1998; Kâ and Hwang, 2011). In addition, a previously reported transcriptome approach detected 1–20 RNA viruses in each invertebrate species (Shi *et al.*, 2016), suggesting the presence of more RNA viruses associated with marine zooplankton. Additionally, high proportions of unclassified sequence data were obtained, including possible full-length viral segments, because FLDS effectively obtained the sequences of dsRNA viruses and replicating intermediates of ssRNA viruses. These possible full-length segments of RNA viruses with no BLASTX hits to known viruses (e-value cut-off: 1×10^–5^) are available in the public database for future studies (see the section of “*Accession numbers*”). To discover a higher number of RNA viruses associated with the zooplankton community, further efforts for sampling and sequencing as well as the development of a viral database, particularly in zooplankton species, are needed.

RNA virus communities have been poorly investigated in marine zooplankton, and we herein revealed unique RNA virospheres, which differed from those of RNA viruses in marine microbes (mostly bacteria and protists) using FLDS (Urayama *et al.*, 2018) and invertebrate species (*e.g.*, Annelida, Arthropoda, Mollusca, and Nematoda) using a conventional transcriptome ana­lysis (Shi *et al.*, 2016). In the present study, the dominant viral groups in ssRNA viruses were narna-like and picorna-like viruses. Although members of *Narnaviridae* mainly infect fungi (Hillman and Cai, 2013), *Narnaviridae* and *Picornavirales* have both been detected as major groups of eukaryotic microbial RNA viruses in the global ocean (Kaneko *et al.*, 2021). *Picornavirales* are diverse, ubiquitous, and abundant in marine ecosystems (Culley *et al.*, 2003, 2006), and infect a number of invertebrates and vertebrates (Le Gall *et al.*, 2008). The predominant *Picornavirales* OTU formed a novel phylogenetic group with viruses from invertebrates (Shi *et al.*, 2016), mainly with insect viruses of the family *Dicistroviridae*. Moreover, the genome structure of this OTU was consistent with that from this family. Some *Dicistroviridae* viruses cause severe diseases (Valles *et al.*, 2017), and the dominance of this virus in sequence reads indicates the active replication of ssRNA viruses, which may have ecological impacts on zooplankton, such as the dominant copepods.

As shown in the global ana­lysis of eukaryotic microbial viruses (Kaneko *et al.*, 2021), partiti-like viruses were the most diverse and abundant dsRNA viruses in the present study. Although established groups of *Partitiviridae* mainly infect plants and fungi (Nibert *et al.*, 2014), partiti-like viruses in the present study were phylogenetically distinct from previously known groups of this family. Partiti-like viruses in the present study formed unique phylogenetic groups with viruses from invertebrates and marine microbes; therefore, a novel virus-host relationship may be associated with zooplankton in *Partitiviridae*. In other major groups of dsRNA viruses, invertebrates or vertebrates are hosts for *Reoviridae*, *Totiviridae*, and *Picobirnaviridae* (Poulos *et al.*, 2006; Haugland *et al.*, 2011; King *et al.*, 2012; Zell *et al.*, 2017). As observed in *Partitiviridae*, these families harbored phylogenetic groups distinct from known viruses, indicating that zooplankton viruses play a key role in the evolution of RNA viruses. Although the genome of *Picobirnaviridae* is bipartite, we detected a possible non-segmented genome in a picobirna-like virus, as previously reported in the marine virome (Urayama *et al.*, 2018). The family *Chrysoviridae* is known to infect fungi (Ghabrial *et al.*, 2018), and the predominant *Chrysoviridae* in the present study was similar to *Alphachrysovirus*, which infects the fungus *Aspergillus fumigatus* (Jamal *et al.*, 2010). We also recovered a full viral genome similar to that of viruses from the *Hypoviridae* family, which are known to infect fungi (Suzuki *et al.*, 2018). Although we cannot exclude the possibility that these viruses are from the microbiomes associated with host species (Shi *et al.*, 2017), chryso-like and hypo-like viruses have been detected in other invertebrates by a transcriptome ana­lysis (Shi *et al.*, 2016), and metazoan zooplankton may be the host of the viruses detected in the present study.

To the best of our knowledge, this is the first study on the RNA virosphere associated with a zooplankton community collected by a plankton net (mesh size: 100‍ ‍μm). It represents the first step for revealing the interactions between zooplankton and viruses, and the following issues need to be resolved in future studies. We observed high proportions of unassembled and unclassified sequences, and the number of viral genomes recovered was limited. Additional sequencing efforts will help to detect a greater diversity of viruses as well as to recover viral genomes associated with zooplankton. The recovery of viral genome sequences from zooplankton will help reveal the phylogenetic relationships of RNA viruses because arthropods including copepods are considered to play a key role in the evolution of RNA viruses (Li *et al.*, 2015) (Chang *et al.*, 2021. Arthropods and the evolution of RNA viruses, *bioRxiv*. doi: 10.1101/2021.05.30.446314). In addition, we obtained no direct evidence of the host species of RNA viruses in the present study. Since zooplankton are known vectors of phytoplankton viruses (Frada *et al.*, 2014), we may have detected RNA viruses derived from the prey or symbionts of zooplankton. Investigations on marine zooplankton using plankton nets and microbes from water samples at the same site will contribute to the detailed prediction and study of virus-host relationships. The ecological impact of each RNA virus on host species also warrants further research because some RNA viruses do not cause any disease in their hosts, even under high viral copies, as reported in insects (Miyazaki *et al.*, 1996; Koyama *et al.*, 2015). Further investigations will provide insights into the diversity, ecological roles, and evolution of RNA viruses associated with zooplankton and lead to a more detailed understanding of marine ecosystems.

## Citation

Hirai, J., Urayama, S., Takaki, Y., Hirai, M., Nagasaki, K., and Nunoura, T. (2022) RNA Virosphere in a Marine Zooplankton Community in the Subtropical Western North Pacific. *Microbes Environ ***37**: ME21066.

https://doi.org/10.1264/jsme2.ME21066

## Supplementary Material

Supplementary Material

## Figures and Tables

**Table 1.** Top 10 operational taxonomic units (OTUs) of zooplankton. The rank, proportion of sequence reads, best-hit species, and sequence identity according to BLAST results are listed for each OTU together with information on its putative host taxon.

Rank	Read (%)	Blast hit species	Identity	Accession	Putative taxon
1	11.34	*Clausocalanus furcatus*	1,666/1,677 (99%)	GU969200.1	Copepoda (Clausocalanidae)
2	8.2	*Delibus* sp.	1,576/1,579 (99%)	JQ911952.1	Copepoda (Paracalanidae)
3	5.06	*Clausocalanus furcatus*	839/918 (91%)	GU969200.1	Copepoda (Calanoida)
4	4.45	*Cosmocalanus darwinii*	1,672/1,702 (98%)	GU969206.1	Copepoda (Calanidae)
5	4.26	*Flaccisagitta enflata*	1,831/1,843 (99%)	DQ351877.1	Chaetognatha (Sagittidae)
6	4.22	*Oikopleura longicauda*	1,769/1,771 (99%)	MK621856.1	Appendicularia (Oikopleuridae)
7	3.55	*Candacia truncata*	1,753/1,754 (99%)	GU969161.1	Copepoda (Candaciidae)
8	3.48	*Triconia borealis*	1,606/1,683 (95%)	MG661033.1	Copepoda (Oncaeidae)
9	3.37	*Triconia borealis*	1,649/1,676 (98%)	MG661033.1	Copepoda (Oncaeidae)
10	3.17	*Oithona atlantica*	1,641/1,646 (99%)	MG661010.1	Copepoda (Oithonidae)
