# Supplementary material for: RNA Virosphere in a Marine Zooplankton Community in the Subtropical Western North Pacific
Source: Microbes Environ. 2022 Jan 1;37(5):ME21066. doi: 10.1264/jsme2.ME21066 (PMC9763039; doi:10.1264/jsme2.ME21066)
Supplement: Supplementary file 1 — Supplementary Material [file 37_21066_s1.pdf]

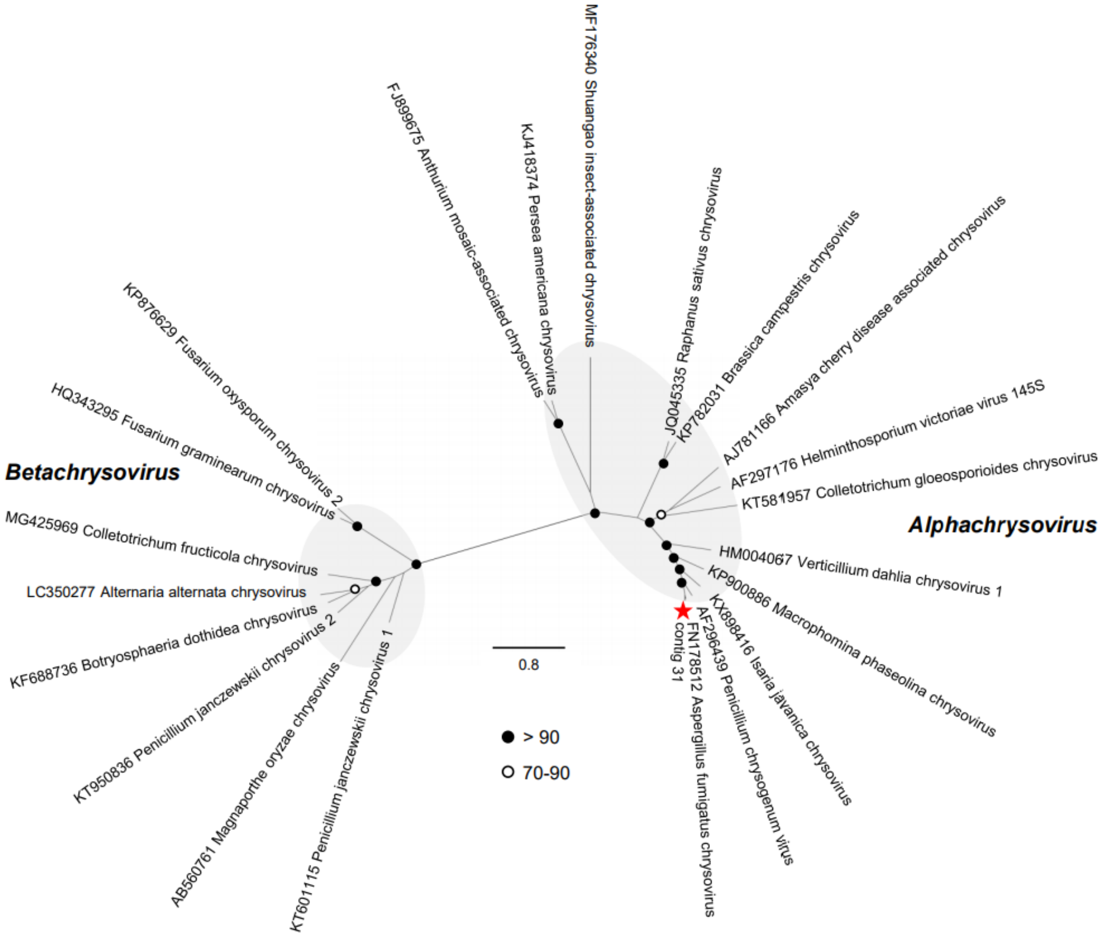

Fig. S1. Maximum likelihood tree for phylogenetic relationships among members of the family *Chrysoviridae* and their relatives using RdRp amino acid sequences. Contigs obtained in this study are represented by red stars. Bootstrap values from maximum likelihood analyses are indicated if ≥ 70%. The best-fitted substitution model was LG+F+G.

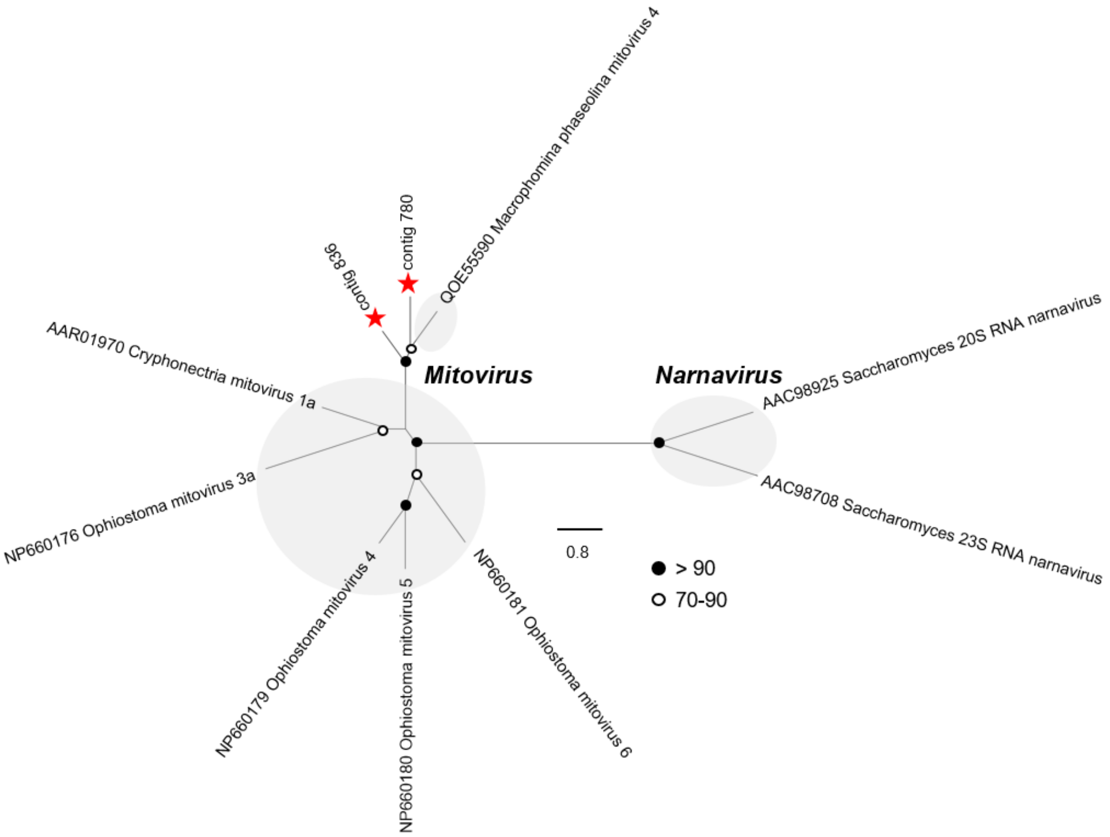

Fig. S2. Maximum likelihood tree for phylogenetic relationships among members of the family *Narnaviridae* and their relatives using RdRp amino acid sequences. Contigs obtained in this study are represented by red stars. Bootstrap values from maximum likelihood analyses are indicated if ≥ 70%. The best-fitted substitution model was LG+F+G.

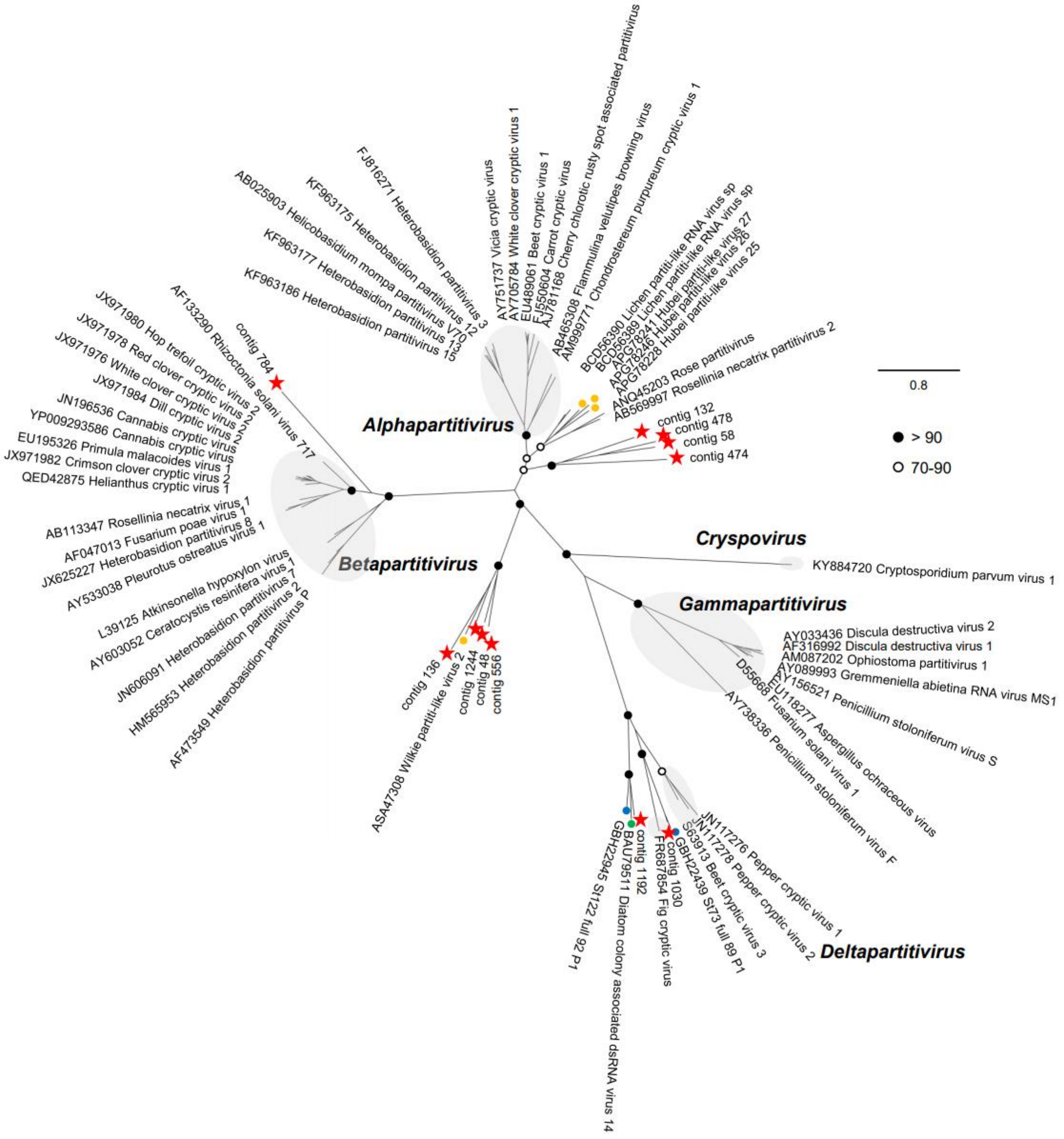

Fig. S3. Maximum likelihood tree for phylogenetic relationships among members of the family *Partitiviridae* and their relatives using RdRp amino acid sequences. Contigs obtained in this study are represented by red stars. Bootstrap values from maximum likelihood analyses are indicated if ≥ 70%. Scale bar indicates genetic distances. The best-fitted substitution model was LG+F+G.

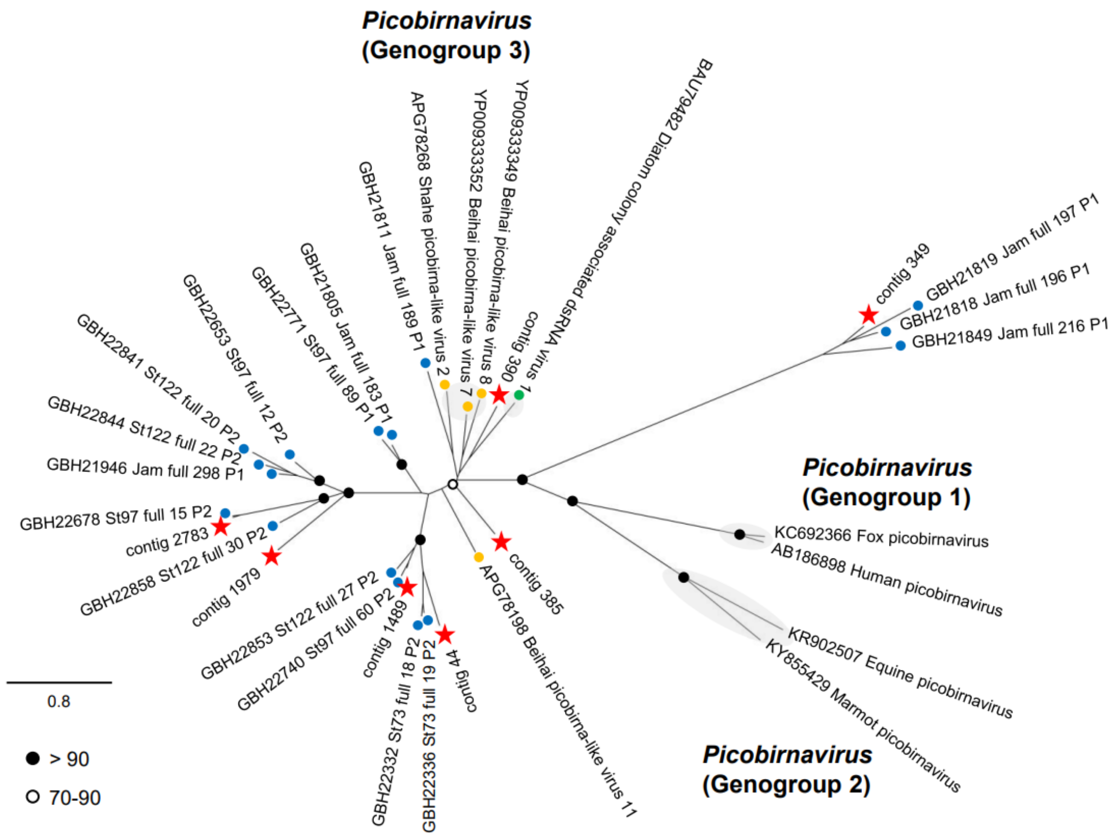

Fig. S4. Maximum likelihood tree for phylogenetic relationships among members of the family *Picobirnaviridae* and their relatives using RdRp amino acid sequences. Contigs obtained in this study are represented by red stars. Bootstrap values from maximum likelihood analyses are indicated if ≥ 70%. Scale bar indicates genetic distances. The best-fitted substitution model was rtREV+F+G.

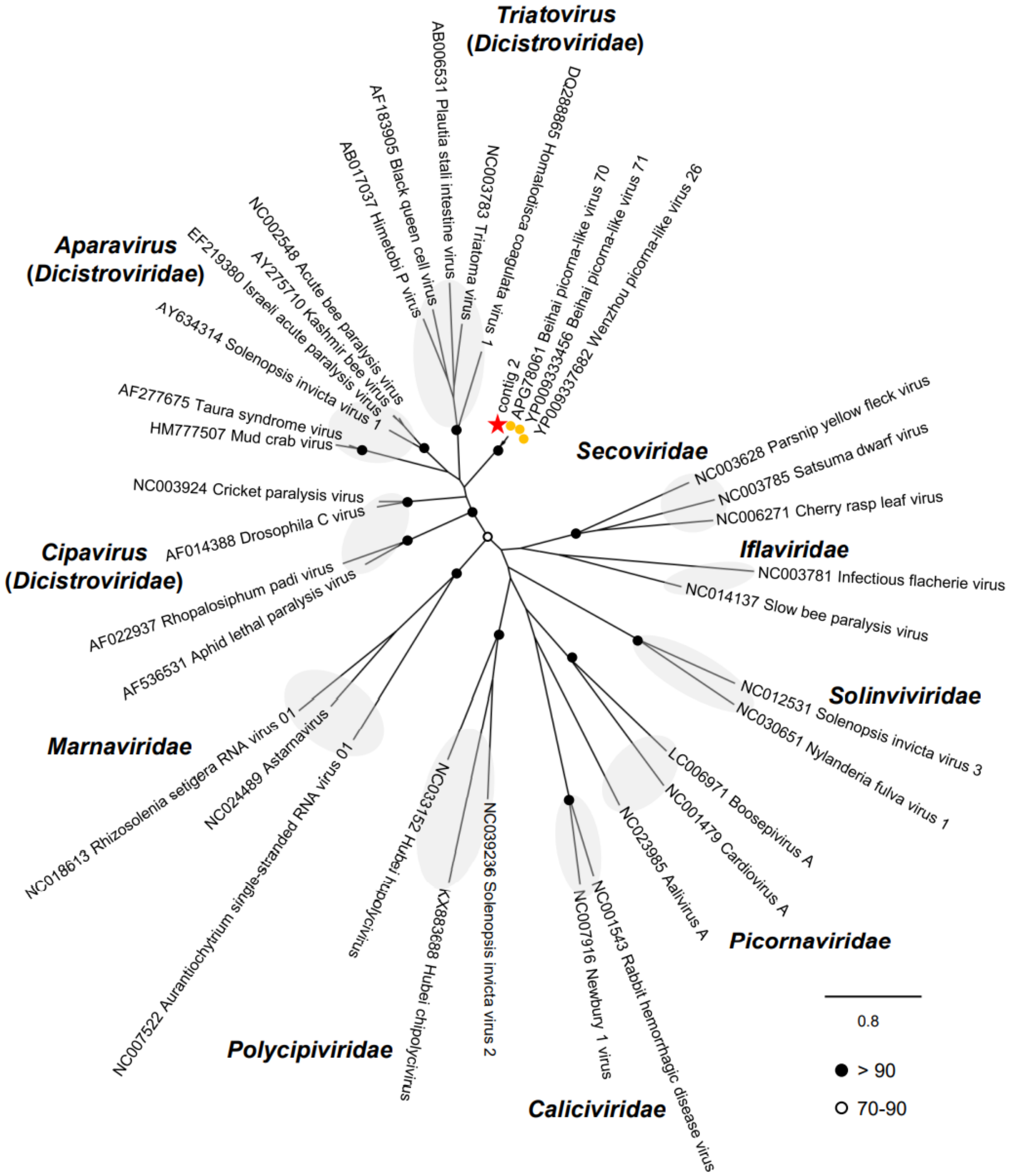

Fig. S5. Maximum likelihood tree for phylogenetic relationships among members of the *Picornavirales* and their relatives using RdRp amino acid sequences. Contigs obtained in this study are represented by red stars. Bootstrap values from maximum likelihood analyses are indicated if ≥ 70%. Scale bar indicates genetic distances. The best-fitted substitution model was rtREV+F+G.

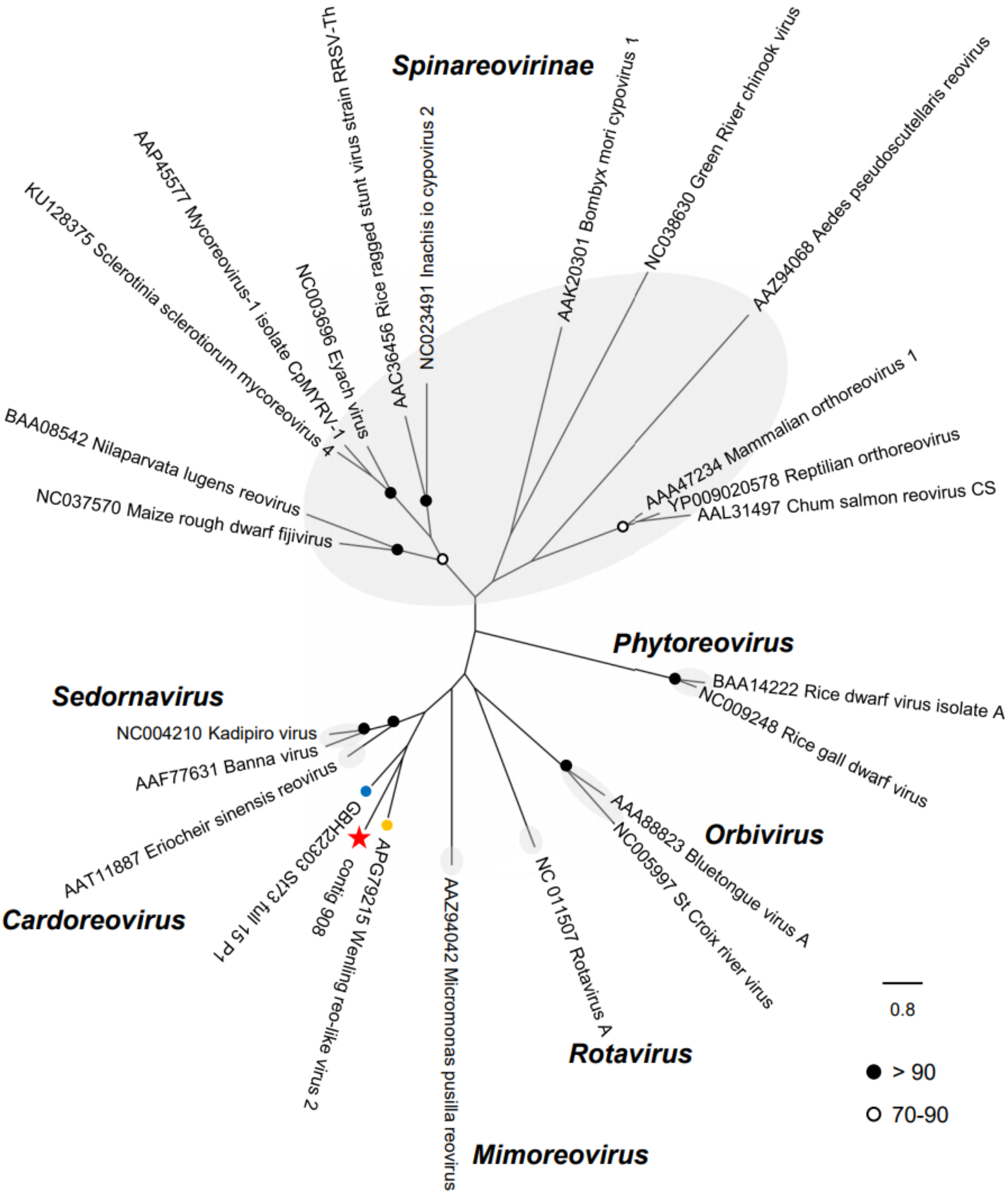

Fig. S6. Maximum likelihood tree for phylogenetic relationships among members of the family *Reoviridae* and their relatives using RdRp amino acid sequences. Contigs obtained in this study are represented by red stars. Bootstrap values from maximum likelihood analyses are indicated if ≥ 70%. Scale bar indicates genetic distances. The best-fitted substitution model was LG4X+F+G.

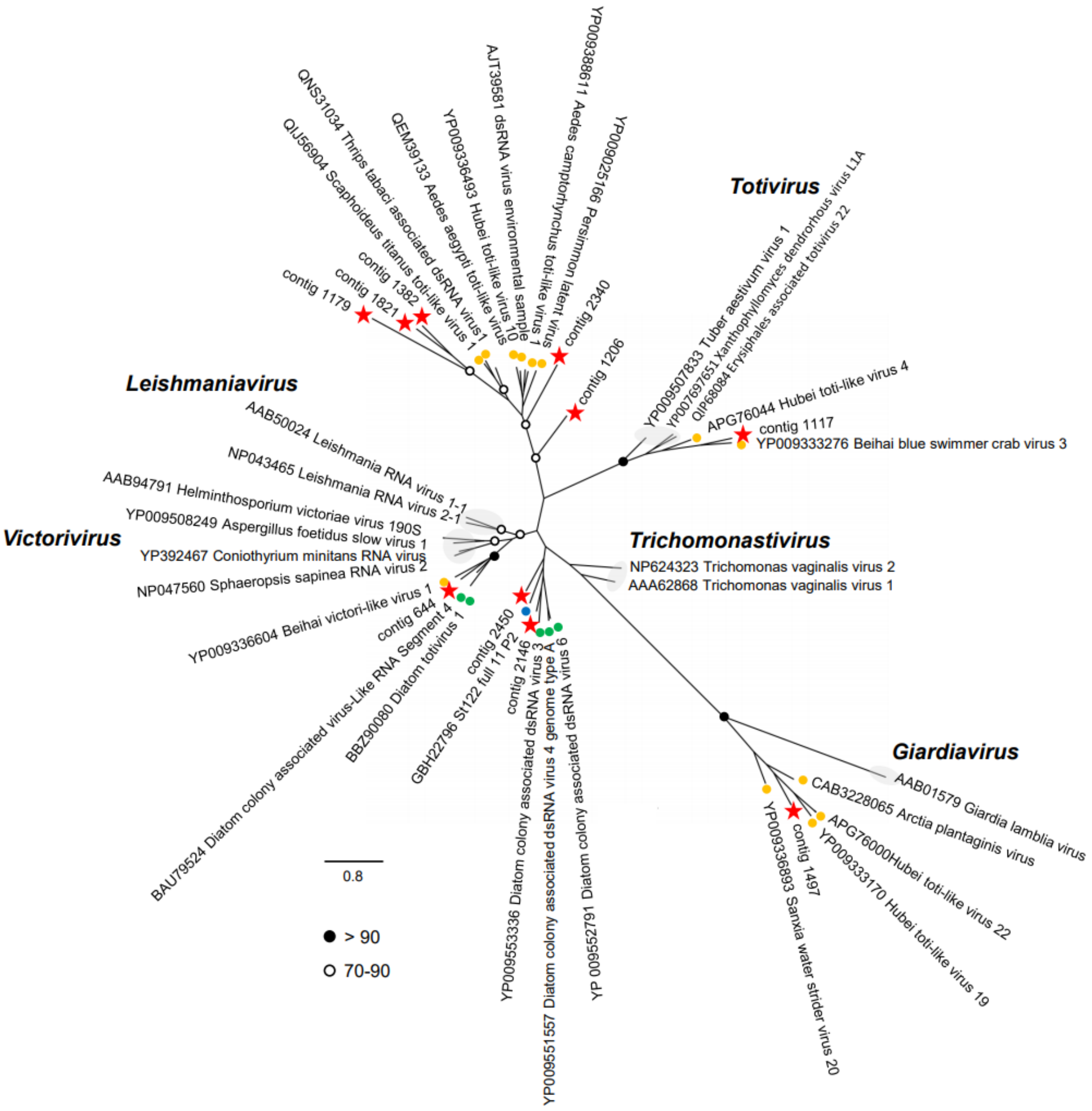

Fig. S7. Maximum likelihood tree for phylogenetic relationships among members of the family *Totiviridae* and their relatives using RdRp amino acid sequences. Contigs obtained in this study are represented by red stars. Bootstrap values from maximum likelihood analyses are indicated if ≥ 70%. Scale bar indicates genetic distances. The best-fitted substitution model was LG+F+G.
